# Bactericidal Activity to *Escherichia coli*: Different Modes of Action of Two 24-Mer Peptides SAAP-148 and OP-145, Both Derived from Human Cathelicidine LL-37

**DOI:** 10.3390/antibiotics12071163

**Published:** 2023-07-08

**Authors:** Ayse Ön, Djenana Vejzovic, James Jennings, Lena Parigger, Robert A. Cordfunke, Jan Wouter Drijfhout, Karl Lohner, Nermina Malanovic

**Affiliations:** 1 Institute of Molecular Biosciences, University of Graz, 8010 Graz, Austria; ayse_oen@hotmail.com (A.Ö.); djenana.vejzovic@medunigraz.at (D.V.); james.jennings@uni-graz.at (J.J.); lena.parigger@edu.uni-graz.at (L.P.); karl.lohner@uni-graz.at (K.L.); 2 Department of Immunology, Leiden University Medical Center, 2333 Leiden, The Netherlands; r.a.cordfunke@lumc.nl (R.A.C.); j.w.drijfhout@lumc.nl (J.W.D.); 3 Field of Excellence BioHealth, University of Graz, 8010 Graz, Austria; 4 BioTechMed Graz, 8010 Graz, Austria

**Keywords:** LPS, lipid–peptide interaction, membrane permeabilization

## Abstract

OP-145 and SAAP-148, two 24-mer antimicrobial peptides derived from human cathelicidin LL-37, exhibit killing efficacy against both Gram-positive and Gram-negative bacteria at comparable peptide concentrations. However, when it comes to the killing activity against *Escherichia coli*, the extent of membrane permeabilization does not align with the observed bactericidal activity. This is the case in living bacteria as well as in model membranes mimicking the *E. coli* cytoplasmic membrane (CM). In order to understand the killing activity of both peptides on a molecular basis, here we studied their mode of action, employing a combination of microbiological and biophysical techniques including differential scanning calorimetry (DSC), zeta potential measurements, and spectroscopic analyses. Various membrane dyes were utilized to monitor the impact of the peptides on bacterial and model membranes. Our findings unveiled distinct binding patterns of the peptides to the bacterial surface and differential permeabilization of the *E. coli* CM, depending on the smooth or rough/deep-rough lipopolysaccharide (LPS) phenotypes of *E. coli* strains. Interestingly, the antimicrobial activity and membrane depolarization were not significantly different in the different LPS phenotypes investigated, suggesting a general mechanism that is independent of LPS. Although the peptides exhibited limited permeabilization of *E. coli* membranes, DSC studies conducted on a mixture of synthetic phosphatidylglycerol/phosphatidylethanolamine/cardiolipin, which mimics the CM of Gram-negative bacteria, clearly demonstrated disruption of lipid chain packing. From these experiments, we conclude that depolarization of the CM and alterations in lipid packing plays a crucial role in the peptides’ bactericidal activity.

## 1. Introduction

The mode of action of antimicrobial peptides (AMPs), a highly conserved set of molecules belonging to the innate first line of defense of various organisms, has been a subject of intense research [1] due to the increasing threat of antimicrobial resistance [2] and dangers associated with bacteria [3,4,5,6]. Initially, a simple concept was proposed that cationic amphipathic AMPs interact with negatively charged membrane lipids, leading to the disruption of the fundamental barrier function of bacterial cells. In addition, various mechanisms of action, such as pore formation, carpet, or detergent mechanisms, have been suggested for AMPs [7,8,9]. Lipidomic studies have identified specific distribution patterns of membrane phospholipids and glycolipids in Gram-positive and Gram-negative bacteria, providing a basis for creating model systems to investigate the mode of action of AMPs and other membrane-active compounds [10,11,12,13]. Typically, at the molecular level, peptides have been more effective in disrupting highly anionic membranes compared to less anionic membranes such as Gram-negative bacteria [14,15,16]. However, in some cases, including the membrane-active compound octenidine (which has structural similarities to peptides), the interaction between the antimicrobial agent and lipids is primarily driven by hydrophobic rather than electrostatic interactions [17,18]. Additionally, some peptides have a larger hydrophobic area, facilitating their insertion into the lipid membrane [15,19]. To better understand the specific interactions between lipids and peptides that lead to efficient bacterial killing, it is necessary to consider not only the nature of the lipid and the structural properties of the peptides [20,21,22,23,24], but also the stability of the entire membrane and the functionality of membrane proteins [25,26,27]. These factors can influence the killing potential of AMPs in vivo.

The AMP Database currently includes over 3000 peptides that are active against both Gram-positive and Gram-negative bacteria [28]. Additionally, included are 625 peptides that are active only against Gram-positive bacteria and 395 peptides that are active only against Gram-negative bacteria. When mapping all frog AMPs, which represent the largest population in the AMP Database, it becomes evident that there is a higher number of peptides active against Gram-positive bacteria [10]. This raises the question of whether peptides are generally less active against Gram-negative bacteria. The fact is that the cytoplasmic membranes (CM) of Gram-negative bacteria are more complex compared to the single membrane of Gram-positive bacteria, as they consist of an outer and an inner cytoplasmic membrane [12]. This complexity makes them more challenging to permeabilize.

Furthermore, the distribution of major phospholipids, such as phosphatidylethanolamine (PE) and phosphatidylglycerol (PG), differs between Gram-negative and Gram-positive bacteria. In Gram-negative membranes, the ratio of zwitterionic to anionic phospholipids is 3:1, whereas in Gram-positive membranes it is 1:3 [12]. It has been suggested that high hydrophobicity may hinder peptide translocation through the outer membrane [29]. Indeed, the majority of frog AMPs are less active or inactive against Gram-negative bacteria when their hydrophobicity content exceeds 51% [10,29]. For example, amphibian temporins [15] have a very high hydrophobic content of 69%, and peptides with lower hydrophobic content, such as thrombocydine TC-19 (40%) [16], human cathelicidin LL-37 (40%), and its derivative OP-145 (46%) [13], exhibit slightly higher potency in killing Gram-positive bacteria. It should be noted that the molecular conformation of an antimicrobial, including recently described lipid-like compounds, is a crucial determinant of membrane activity against both Gram-negative and Gram-positive bacteria [30]. However, the general observation suggests that there is a certain degree of permeabilization for zwitterionic membranes, while a clear preference for anionic membranes is often observed.

Interestingly, OP-145 is a notable exception. Although its antimicrobial activity is in the very low micromolar range, it is not capable of disrupting membranes isolated from *Escherichia coli*, and it does not show significant permeability of the *E. coli* cytoplasmic membrane at concentrations where it effectively kills bacteria [14]. Another LL-37 derivative, SAAP-148, which is one of the most potent AMPs currently known, permeabilizes both anionic and less anionic membranes in model systems and living bacteria, but it exhibits stronger effects on anionic membranes in model systems [13,14,19]. However, when looking at its antimicrobial profile across different Gram-negative bacteria, its activity ranges between 0.8 to 12.8 µM. These observations led to the assumption that differences in antimicrobial activities might correspond to differences in the structure of lipopolysaccharide (LPS) in the outer membrane of Gram-negative bacteria, which could significantly impact interactions with peptides. Recent studies have shown that SAAP-148 can completely screen the surface charges of *E. coli* [14], supporting the idea that LPS may indeed be an important factor influencing the activity of peptides.

The hypothesis becomes even more plausible and experimentally feasible in the case of OP-145, as our previous experiments have shown that it does not permeabilize the membrane but instead neutralizes the surface charge of *E. coli*, indicating an electrostatic binding to the bacterial surface [14]. Despite this lack of membrane permeability, OP-145 exhibits significant bactericidal activity against *E. coli* [14], as well as other Gram-negative bacteria [31] and Gram-positive bacteria [13,31,32]. Moreover, it demonstrates strong interactions with membrane phospholipids [19,32] and LPS [31]. Therefore, it raises the question of whether the interaction with LPS in the outermost layer of *E. coli* (i) prevents OP-145 from penetrating the cytoplasmic membrane and functioning there, (ii) if this interaction with LPS alone is solely responsible for bacterial killing, or (iii) if there are other targets or events involved in the bactericidal activity of OP-145. In the current study, the effect of the peptides SAAP-148 and OP-145 on the membranes of *E. coli* strains producing different lengths of LPS was investigated. Bacteria with varying LPS lengths were treated with peptides, and subsequent changes in membrane properties were analyzed. This analysis aimed to understand how the peptides interact with and affect the membranes of *E. coli* strains with different LPS lengths, providing insights into their mode of action and potential differences in their antimicrobial activities.

## 2. Results

### 2.1. Effect of the Peptides on Binding to Bacterial Surfaces of E. coli Producing Different LPS Lengths 

To investigate whether OP-145 and SAAP-148 exhibit differences in killing *E. coli* when the LPS is altered, we conducted experiments using different *E. coli* strains. These included the FDA-approved strain ATCC25992 with a smooth phenotype (full length), a K12 strain lacking the O-antigen (rough phenotype) and D21f2, which lacks the O-antigen and the outer/inner core of LPS (deep-rough phenotype, containing only Lipid A and 2-Keto-3-desoxy-octonat (KDO) units). We also included strain D21 as a control, which is a K12-derived strain decorated with phosphorylethanolamine in the LPS outer core region [33,34]. A detailed genotype of the mentioned strains is described in the method section (Table 1). Interestingly, both OP-145 and SAAP-148 inhibited the growth of all tested strains at the same minimum inhibitory concentration (MIC) as observed for the smooth-phenotype ATCC25992 strain (Table 2). Moreover, the killing of all tested bacteria occurred at nearly the same peptide concentration. The small up to two-fold difference in LC_99.9%_ (the concentration required for 99.9% killing) indicates a relatively minor variation in the killing capability of the peptides. It is also worth mentioning that <two-fold difference in antimicrobial activity may not always be considered significant, as small errors in determining the cell number in the starting inoculum of the bacterial culture can influence the outcome of antimicrobial susceptibility testing [35]. However, our findings suggest that the presence or absence of certain components of LPS, such as the O-antigen and outer/inner core, does not significantly affect the antimicrobial activity of OP-145 and SAAP-148 against *E. coli.* Both peptides exhibit potent antimicrobial activity against *E. coli* strains with different LPS phenotypes, indicating that their killing mechanism is likely independent of LPS composition. 

**Figure 1:**
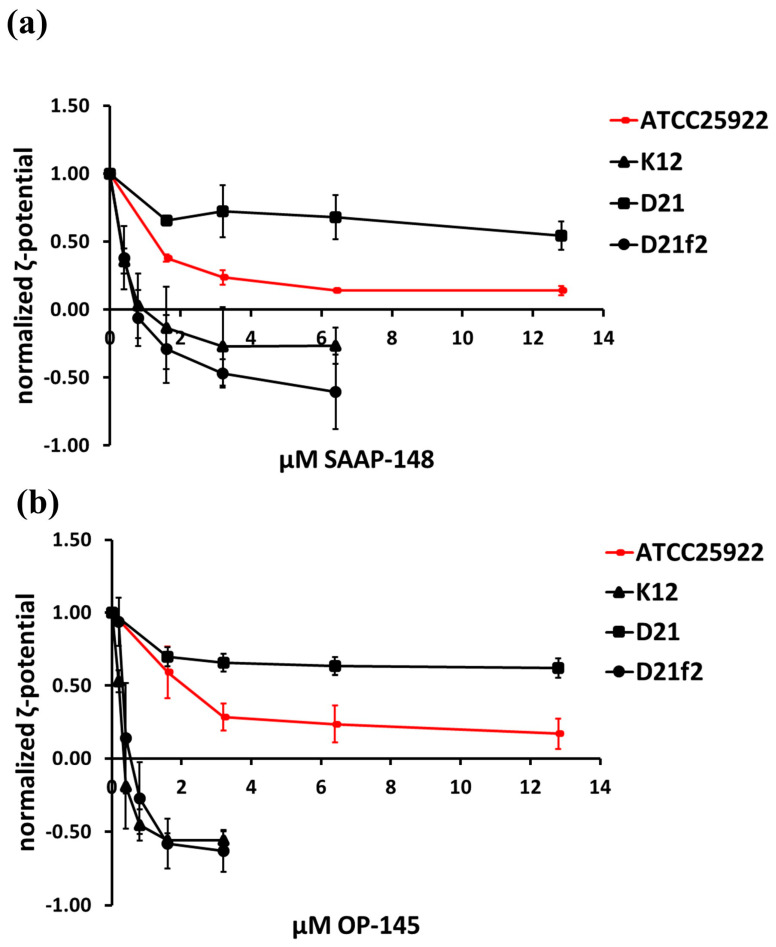
Surface charge neutralization of *E. coli* producing different lengths of LPS induced by SAAP-148 (**a**) and OP-145 (**b**). The bacteria were treated with the peptides at concentrations ranging from 0.2–12.8 µM and incubated for 5 min in Hepes buffer. The surface charge of the bacteria was then measured using a Zetasizer instrument. The zeta potential, expressed in millivolts (mV), was normalized to values measured in the absence of peptides. The results are means of at least three or more independent experiments.

In order to investigate the binding of OP-145 and SAAP-148 to bacteria with truncated LPS, we conducted zeta potential measurements to assess surface charge neutralization. The results showed that both peptides completely neutralize the surface charge of the smooth phenotype strain. However, in contrast to the rough and deep-rough phenotypes in the K12 and D21f2 strains, the peptides did not overcompensate the surface charge (Figure 1). This suggests that the peptides remain bound to the bacterial surface. Interestingly, there was no significant difference observed between the strain lacking only the O-antigen and the strain lacking both the O-antigen and the entire core region. This indicates that the binding of both peptides to the outermost layer of the bacterial cell envelope is crucial for initiating the killing process. The binding pattern observed in the D21 strain, which has a different decoration in the core region, followed a similar trend to that of the ATCC25992 strain. These findings suggest that the specific binding characteristics of the peptides to different LPS phenotypes and core decorations may influence their overall effectiveness.

### 2.2. Effect of the Peptides on Membranes of E. coli Producing Different LPS Length 

**Figure 2:**
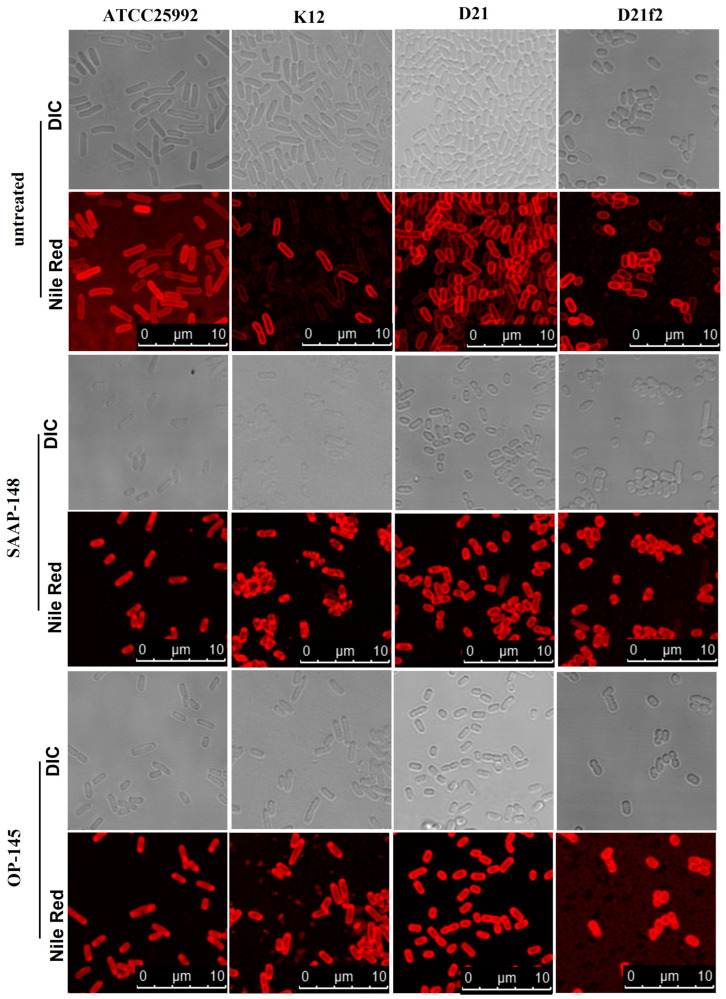
Membrane staining of *E. coli* producing different lengths of LPS by SAAP-148 and OP-145. Bacteria were treated with peptides at twice their MIC, with concentrations of 3.2 µM for SAAP-148 and 12.8 µM for OP-145. After treatment, bacteria were stained with Nile Red, and both fluorescence and bright-field images were captured for all samples including untreated bacteria. The pictures were captured from three independent experiments and only representatives are shown.

To detect changes in membranes, the bacteria were stained with the fluorescent membrane dye Nile Red, which is sensitive to the polarity and fluidity of a membrane (Figure 2). In the untreated bacteria, Nile Red staining of membranes appeared nearly homogeneous. However, when exposed to both SAAP-148 and OP-145, bacteria producing smooth and rough/deep rough LPS showed a similar pattern of intense staining areas or foci around the poles. This observation suggests that a similar process is occurring in both types of bacteria, independent of the LPS composition. Furtheremore, this phenomenon has also been observed with both peptides in *Enterococcus hirae* [19] and with octenidine in *E. hirae* [17], *Bacillus subtilis* [17], and *E.coli* [18]. These staining patterns likely indicate irregularities in the membrane and are likely associated with changes in membrane fluidity.

**Figure 3:**
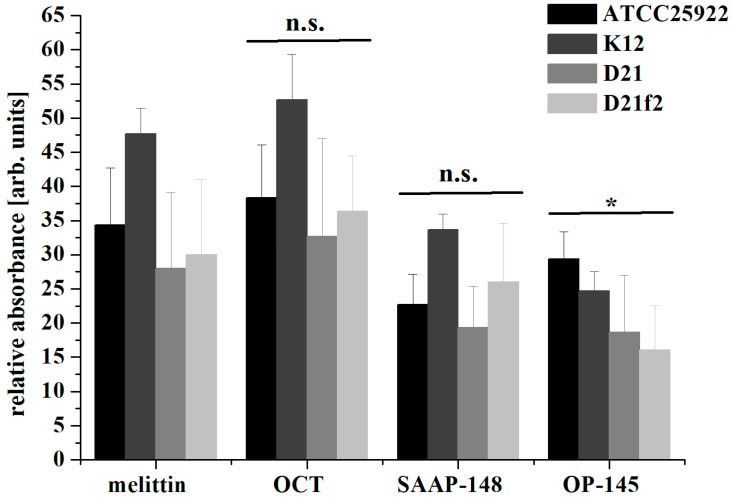
Membrane depolarization of *E. coli* producing different lengths of LPS by SAAP-148 and OP-145. Bacteria were treated with 0.001% OCT, 3.65 µM Melittin or 0.4 µM peptides SAAP-148 and OP-145. Treated bacteria were stained with DiSC3(5), and the fluorescence was normalized to untreated bacteria. The results are means of at least three or more independent experiments. Significance of the difference between melittin as a control and peptides was calculated with unpaired two-sided *t*-test considering 95% confidence intervals (*p* ≤ 0.05). n.s. was considered not significant for *p* > 0.05 and * was considered as significant with *p* ≤ 0.05.

**Figure 4:**
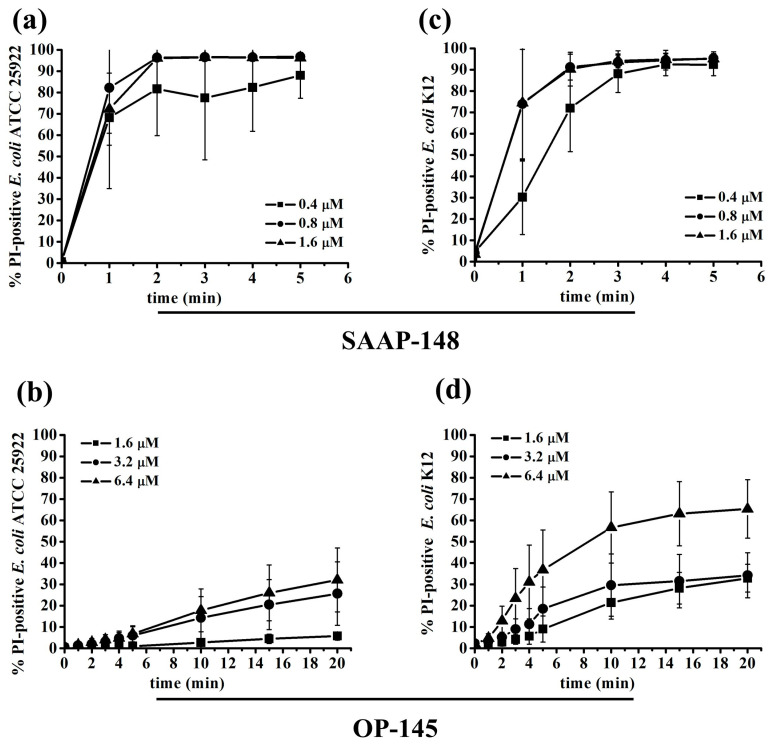
Permeabilization of bacterial membranes by SAAP-148 and OP-145. *E. coli* ATCC25992 (**a**,**b**) and K12 (**c**,**d**) labeled with PI and exposed to different peptide concentrations including sub-MIC, MIC and above the MIC. The influx of PI was monitored by measuring the PI fluorescence using FACs at durations of 5–20 min. The percentage of PI-positive *E. coli* cells after the addition of 1.6 to 6.4 µM OP-145 and 0.4 to 1.6 µM SAAP-148 was calculated at various time intervals. The results are means of at least three or more independent experiments.

**Figure 5:**
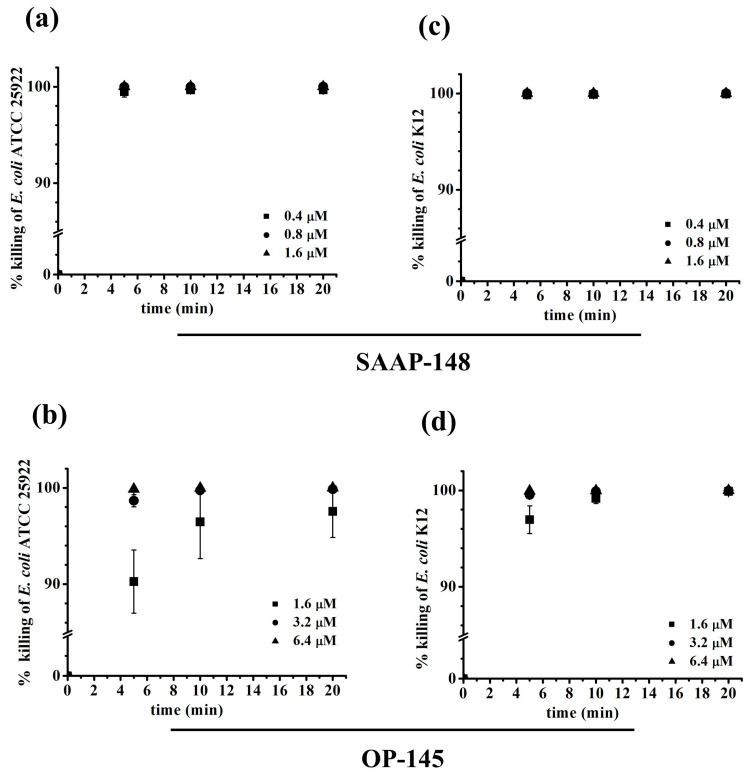
The killing of bacteria by SAAP-148 and OP-145. *E. coli* ATCC25992 (**a**,**b**) and K12 (**c**,**d**) were exposed to various concentrations of peptides including sub-MIC, MIC and above the MIC. At different time intervals (5, 10, and 20 min), treated bacteria were plated on sensitive diagnostic agar. After 24 h, bacterial colonies were counted, and the percentage of surviving *E. coli* cells was calculated for each time interval. This calculation was based on the number of colonies observed compared to the initial bacterial population, after exposure to concentrations ranging from 1.6 to 6.4 µM of OP-145 and 0.4 to 1.6 µM of SAAP-148. The results are means of at least three or more independent experiments.

To further investigate membrane damage and disruption, we conducted membrane depolarization assays using the voltage-sensitive fluorescent dye DiSC3(5) (Figure 3), and performed flow cytometry experiments with the membrane-impermeable fluorescent dye propidium iodide (PI) to quantify the extent of membrane damage. PI serves as an indicator for membrane disruption and only permeabilizes severely damaged membranes. The increased fluorescence of DiSC3(5) indicates membrane depolarization, which is associated with small perturbations in the membrane. Both SAAP-148 and OP-145 induced at least 70% of the DiSC3(5) signal compared to the control peptides melittin and octenidine in all tested strains. Notably, OP-145 showed a slight decrease in membrane depolarization in the rough LPS strain (K12) compared to the smooth LPS strain (ATCC25992), while this was not observed with other compounds. This observation suggests that the absence of the outermost layer in bacterial envelopes, as seen in the rough LPS strain, may increase membrane permeability. Using PI uptake as an indicator of severe membrane disruption, we found that at a concentration of 1.6 µM, OP-145 did not permeabilize membranes in *E. coli* expressing smooth LPS, even though more than 90% of bacteria were dead (Figure 4b and Figure 5b). Interestingly, higher concentrations of OP-145 increased membrane permeability up to 30%, indicating some level of membrane damage occurring after cell death (Figure 4d and Figure 5d). In contrast, nearly 100% of cells exposed to SAAP-148 in the smooth LPS strain showed membrane permeability (Figure 4a and Figure 5a), while in the rough LPS strain, membrane permeability slightly decreased without affecting cell viability (Figure 4b and Figure 5b).

### 2.3. Effect of the Peptides on Phospholipids

**Figure 6:**
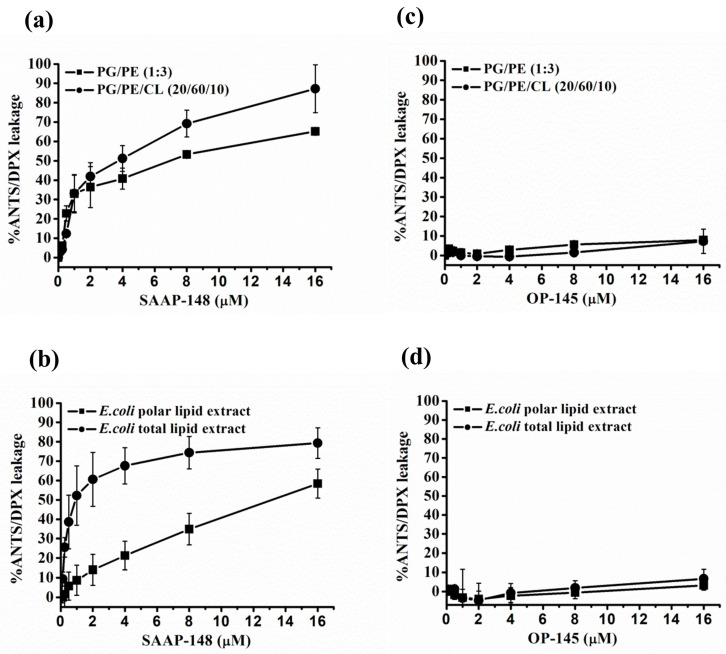
Permeability of model membranes by SAAP-148 and OP-145. The leakage of ANTS was measured from LUVs composed of synthetic phospholipids Palmitoyl-oleoyl phosphatidylglycerol (POPG), Palmitoyl-oleoyl-phosphatidylethanolamine (POPE), and Tetraoleoyl-cardiolipin (TOCL) (**a**,**b**) or lipids isolated from *E. coli* (**c**,**d**). The fluorescence of ANTS was monitored as an indicator of its release upon titration with the indicated concentrations of OP-145 and SAAP-148. The results are means of at least two or more independent experiments performed in duplicate.

In our previous experiments, SAAP-148 and OP-145 were found to cause limited or no disruption of membranes composed of lipids isolated from *E. coli* in a polar lipid fraction [12]. This observation was consistent with membranes composed of PE/PG, where disruption was minimal or absent, whereas membranes composed solely of PG were significantly disrupted even at lower concentrations [13,14,19,32]. However, the lack of permeabilization in PE-containing membranes does not explain the significant membrane permeabilization observed here in *E. coli* strains with smooth and rough LPS (Figure 5). Lipid analysis revealed that smooth and rough LPS strains had similar membrane lipid compositions, with major lipid species identified as PE, PG, and CL in the lipid polar extracts [42]. This prompted us to investigate if the peptides interact specifically with other targets such as CL, LPS, or phospholipids with different saturation levels, which could enhance their membrane activity. Leakage experiments were conducted using large unilamellar vesicles (LUVs) containing total lipid extracts from *E. coli* or synthetic phospholipids containing PE/PG and CL, with the entrapment of ANTS/DPX as indicators. When comparing PG/PE LUVs with and without CL (Figure 6a,c), no significant changes were observed for OP-145, while a slight increase in membrane permeabilization was seen for SAAP-148. Similarly, when comparing total lipid extracts to polar lipid extracts, no differences in leakage were observed for OP-145, whereas a marked increase in ANTS release from 10 to 60% was detected at 1–2 µM SAAP-148 (Figure 6b,d). These findings strongly suggest that other targets, most likely the major constituent of *E. coli* membranes LPS, might potentiate the membrane activity of SAAP-148.

**Figure 7:**
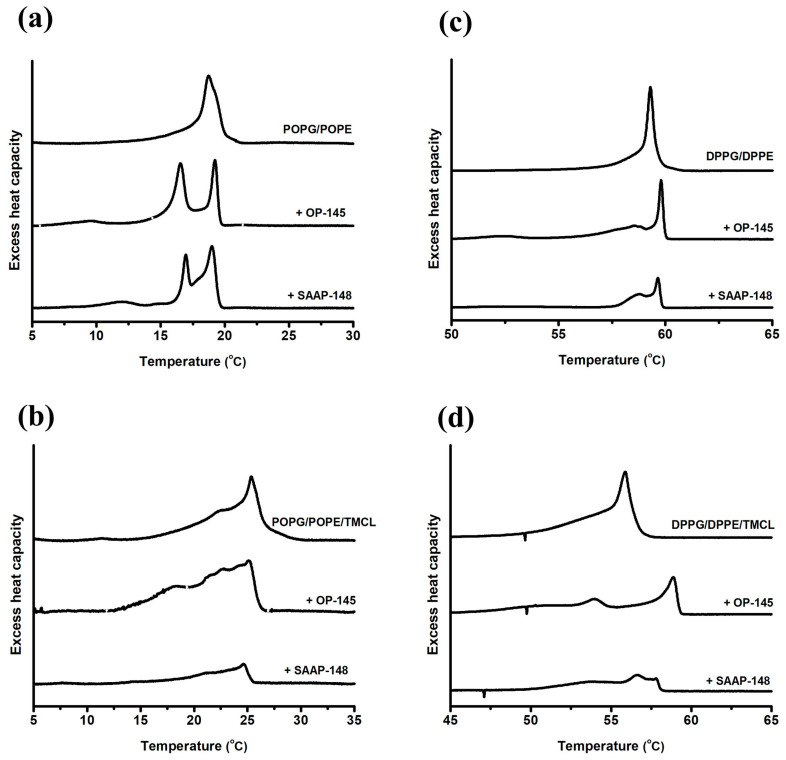
Thermotropic behavior of model membranes exposed to OP-145 and SAAP-148. DSC cooling scans of membranes composed of unsaturated POPG/POPE (**a**), POPG/POPE/TMCL (**b**) and saturated DPPG/DPPE (**c**) and DPPG/DPPE/TMCL (**d**) in presence and absence of SAAP-148 and OP-145. DSC scans were recorded at 1 mg/mL membrane with a lipid-to-peptide molar ratio of 25:1. In contrast to membranes composed of saturated fatty acids in DPPG/DPPE, membranes containing unsaturated fatty acids in POPG/POPE exhibit a lower main transition temperature (*T*_m_) and a lower enthalpy. The half-width of transition (∆*T*_1/2_), however, increases due to lower cooperativity. For reference, pure POPG has a main transition temperature around −5 °C [32,43], POPE at about 24 °C [44,45], DPPG [32] at 41.5 °C, DPPE at 63.4 °C [17,18] and TMCL at 17.9 °C, 27.9 °C and 41 °C [19]. The experiments have been performed in triplicate and only representative scans are shown.

To investigate the influence of fatty acid composition on interactions of the peptides with phospholipids (PLs), we conducted differential scanning calorimetry (DSC) experiments on PG/PE membranes with different fatty acyl species (saturated and/or unsaturated) (Figure 7, Table 3). Specifically, we compared PG/PE membranes esterified with palmitoyl (P) and oleoyl (O) fatty acyl chains at the sn-1 and sn-2 positions (representing unsaturated membranes, POPG/POPE) with PG/PE membranes esterified with palmitoyl fatty acid chains at both sn-1 and sn-2 positions (representing saturated PG/PE, DPPG/DPPE). The DSC scans revealed the thermotropic phase behavior of both POPG/POPE (Figure 7a) and DPPG/DPPE (Figure 7c), characterized by a single-phase transition from the gel phase to the fluid phase. POPG/POPE exhibited a main transition at 21.1 °C, while DPPG/DPPE had a main transition at 60.4 °C. Notably, a significant shift in the main transition temperature was observed upon cooling (Figure 7a,c and Table 3). In the presence of the peptides, a disordering effect on both PG/PE model membranes was evident, as observed by the formation of domains melting at different temperatures. The phase transitions induced by the peptides occurred both below and above the main phase transition temperature of the respective model membranes. It is expected that domains with a higher PE content would shift to higher phase transition temperatures, while domains with an increasing PG content would shift to lower phase transition temperatures. Since both peptides carry a positive charge, they are more likely to interact with the negatively charged PG, leading to enrichment in these domains. This observation is consistent with previous evidence that both peptides can disrupt PG membranes [13,14,19,32]. Additionally, an increase in total enthalpy was observed for both peptides and both model membranes. It has been previously reported that an increase in phase transition temperature and enthalpy, as observed for OP-145 and SAAP-148, could be associated with tighter lipid packing when peptides induce “quasi-interdigitation” in a bilayer [13,19,32]. However, due to the existence of overlapping domains, accurate determination of enthalpies and specific effects is challenging. This is further complicated when cardiolipin (CL) (specifically, 1,1′,2,2′-myristoyl CL, TMCL) was incorporated into the PG/PE membranes (Figure 7b,d, Table 3). Although the peptides may not significantly disrupt membranes containing CL or PE, it is evident that they both exhibit significant interactions with membranes containing PG or PE species. These interactions can be associated with membrane damage or, at the very least, destabilization of the membrane in living bacteria.

## 3. Discussion

Determining the factors that contribute to the function of AMPs and identifying their dominant properties is always a question of interest. In our previous experiments, we conducted a detailed comparison of the physicochemical properties of OP-145 and SAAP-148, as well as their effects on bacterial membranes [14,19]. The main difference between the peptides lies in their overall charge, with SAAP-148 having a charge of +11 compared to +6 for OP-145. However, the length of 24 amino acid residues and the total hydrophobicity remain the same between the two peptides. Interestingly, the most noticeable difference between the peptides was observed in terms of membrane permeability at both the molecular and cellular levels. SAAP-148 demonstrated superiority over OP-145, and this difference was not solely driven by electrostatic interactions. We found that in vesicles composed solely of PG, the complete coverage of surface charges by both peptides resulted in complete membrane disruption (100% membrane leakage) [14]. However, when membranes contained PE in PG/PE or *E. coli* lipid extract, full coverage of surface charges by SAAP-148 caused only 5–20% membrane leakage, while OP-145 did not induce any permeabilization [14]. Furthermore, even in membranes composed of PG/CL, which mimic the cytoplasmic membrane of Gram-positive bacteria such as *Enterococcus hirae*, no electrostatic interaction during membrane disruption was observed [19]. Based on these findings, we concluded that the larger hydrophobic region of SAAP-148 might be responsible for its superior activity, facilitating stronger partitioning and insertion into the membrane. Additionally, the presence of W5 and W16 residues in SAAP-148, which interact with the membrane interface but are absent in the OP-145 sequence, further supports stronger insertion of SAAP-148 into the membrane. The interaction between Q18 and Y15 in the hydrophobic region with CL head groups might also play a role, as these amino acid residues contain additional amide or hydroxy groups that promote hydrogen bonding [46]. This interaction may enhance the binding of SAAP-148 to phospholipids like CL and PE, which are also capable of forming hydrogen bonds [10,47]. Furthermore, this observation could explain why SAAP-148 is more efficient in disrupting membranes containing PE, such as the *E. coli* membrane [11]. On top of that, our DSC studies revealed that OP-145 exhibits notable phase separation in different model membranes including PG [32], PG/CL [19], and PG/PE/CL (as shown in Figure 7). Phase separation of phospholipids and peptides occurs when they segregate into separate lipid-rich and peptide-rich domains within a membrane. This can result in reduced interaction between the two components, leading to less pronounced effects on membrane properties [48].

Although the peptides, in particular OP-145, may not cause significant disruption to membranes composed of PE, it is clear that they interact markedly with membranes containing PE species. This interaction is also reflected by the induction of irregularities (membrane depolarization, Nile Red staining) in the membrane at the cellular level. In the case of SAAP-148, these irregularities are associated with substantial membrane damage and permeabilization. The results obtained from the membrane staining and depolarization assay indicate that OP-145 induces membrane damage to a certain extent, without causing significant membrane permeabilization. For OP-145, the observed irregularities in the membrane can indeed play a critical role in destabilizing the membrane and compromising its vital barrier function. As these irregularities progress, the membrane starts to lose its integrity, resulting in cellular damage and eventually leading to cell death. Indeed, when bacteria are incubated with OP-145 for a longer period, there is a significant increase in membrane permeability. The same was observed when peptides were exposed to bacteria that did not express O-antigen in the outermost LPS layer. However, our findings indicate that the presence or absence of certain components of LPS, such as the O-antigen and outer/inner core, does not significantly affect the antimicrobial activity of OP-145 and SAAP-148 against *E. coli*. These findings suggest that the killing mechanism of these peptides is likely to be independent of LPS composition. While LPS can contribute to the overall charge [42] and stability of the bacterial membrane [49], it cannot be the sole determinant of interactions between peptides SAAP-148 and OP-145 and the membrane. It is important to note that the stability of the LPS can vary among different strains of *E. coli* or Gram-negative bacteria and other factors [49,50]. Changes in LPS stability can impact the susceptibility of the bacterial membrane to AMPs [49] and other antimicrobial agents such as colistin [51]. Interestingly, in our study, the presence or absence of specific LPS components did not significantly affect the antimicrobial activity of OP-145 and SAAP-148 against *E. coli*, indicating that these peptides have a primary killing mechanism that is independent of LPS composition. However, it cannot be excluded that the binding of peptides to the bacterial surface, including all different LPS phenotypes, can initiate downstream events leading to disruption of cytoplasmic membrane integrity and bacterial death. In addition, these binding interactions could also play a role in the peptides’ ability to neutralize LPS and modulate an immune response. It is important to note that the immunomodulatory activities of AMPs such as OP-145 are well-documented [31], and their binding to LPS can contribute to these activities. In this regard, the significant difference in bacterial surface neutralization between smooth and rough phenotypes, as depicted in Figure 1, is noteworthy. This observation suggests that the majority of peptide molecules bind to the O-antigen. This finding is also supported by the increased permeability of the cytoplasmic membrane caused by the peptides, indicating that a larger pool of peptide molecules is available for interactions with the membrane. Also, studies on other AMPs like lactoferricin derivatives indicated that a minor fraction of the total number of AMPs binds to bacterial envelopes and targets mainly inner bacterial components [52]. Consequently, it is less likely that the high hydrophobicity exhibited by both peptides hinders their translocation to the cytoplasmic membrane. Nevertheless, it is possible that the specific binding characteristics of the peptides to different LPS phenotypes and core decorations may influence their overall effectiveness, but they are not the primary cause of bacterial killing. Further studies are necessary to elucidate the specific mechanisms and contributions of these interactions in the antimicrobial and immunomodulatory activities of these peptides. Although both peptides are alpha helical [32,53], further studies are needed to elucidate differences in amphipathicity and stability of alpha helices with respect to membrane–peptide interactions.

In fact, the results obtained with OP-145 lead to the conclusion that inducing membrane irregularities, particularly through membrane depolarization, effectively destabilizes the bacterial membrane and impairs the vital barrier function of a bacterial cell. The gradual increase in membrane permeability over time highlights the progressive nature of the interaction between OP-145 and the bacterial membrane. This also suggests that OP-145 acts over a prolonged period to destabilize the membrane and compromise its integrity, eventually leading to the demise of the bacterial cell.

The mechanism by which the AMPs SAAP-148 and OP-145, derived from human cathelicidin LL-37, act against *E. coli* can be described as follows: (i) interaction with the bacterial surface: SAAP-148 and OP-145 bind to the surface of *E. coli*, likely through electrostatic interactions between the positively charged peptides and negatively charged components of the bacterial surface. (ii) Disruption of the cytoplasmic membrane: although the extent of membrane permeabilization may not directly correlate with their bactericidal activity, both SAAP-148 and OP-145 have the ability to disrupt the integrity of the *E. coli* cytoplasmic membrane. This disruption leads to an increase in membrane permeability, causing leakage of cellular contents and disruption of the ion balance. (iii) Perturbation of lipid packing: studies using model membranes that mimic the composition of Gram-negative bacteria, including *E. coli*, have shown that SAAP-148 and OP-145 induce disordering of the lipid chain packing. This perturbation of lipid packing contributes to depolarization of the cytoplasmic membrane, which is a crucial step in the antimicrobial activity of these peptides. (iv) LPS-independent mechanism: Interestingly, the antimicrobial effects and membrane depolarization caused by SAAP-148 and OP-145 are not significantly influenced by the presence of different LPS phenotypes in *E. coli* strains. This suggests that the mode of action of these peptides is not importantly dependent on interactions with LPS but involves additional factors. Indeed, the focus on a specific cell type, *E. coli*, limits the generalizability of these findings to other organisms. The study’s concentration on the smooth or rough/deep-rough LPS phenotypes of *E. coli* strains may also restrict the understanding of the peptides’ effects on strains that completely lack LPS. Examining the impact of the peptides on such LPS-deficient strains could provide additional insights into the influence of LPS on the peptides’ binding patterns and membrane permeabilization effects. Therefore, this limitation should be acknowledged, and further studies on a broader range of bacterial species and LPS-deficient strains would enhance understanding of the peptides’ mode of action.

In summary, the mode of action of SAAP-148 and OP-145 in *E. coli* involves their interaction with the bacterial surface, disruption of the cytoplasmic membrane, perturbation of lipid packing, and subsequent depolarization of the membrane. These combined actions contribute to the antimicrobial activity exhibited by these peptides.

## 4. Materials and Methods

### 4.1. Peptides

OP-145 (acetyl-IGKEFKRIVERIKRFLRELVRPLR-amide) and SAAP-148 (acetyl-LKRVWKRVFKLLKRYWRQLKKPVR-amide) were synthesized as previously described [13,32]. The purity of the peptides, determined by UPLC-MS (Acquity, Waters, Milford, MA, USA), was >95%, their identity was confirmed with MALDI-TOF mass spectrometry (Microflex, Bruker, Bremen, Germany). The peptides were stored at −20 °C until use, then dissolved in 0.1 % acetic acid pH 3.3 to a stock of 10 mg/mL. Aliquots were also stored at −20 °C. 

### 4.2. Lipids

All phospholipids (>99% purity) were obtained from Avanti Polar Lipids (Alabaster, AL) and stored at −20 °C before usage. The synthetic phospholipids used in this study are DPPG (1,2-dipalmitoyl-sn-glycero-3-[phospho-rac-(1-glycerol)]), DPPE (1,2-dipalmitoyl-sn-glycero-3-phosphoethanolamine), TMCL (1,1′,2,2′-tetramyristoyl cardiolipin,), POPG (1-palmitoyl-2-oleoyl-sn-glycero-3-[phospho-rac-(1-glycerol)], and POPE (1-palmitoyl-2-oleoyl-sn-glycero-3-phosphoethanolamine). In addition, *E. coli* Polar Lipid Extract with a phospholipid content of PE 67%, PG 23.2%, and CL 9.8% as well as *E. coli* Total Lipid Extract with a phospholipid content of PE 57.5%, PG 15.1%, CL 9.8%, and unknown 17.6% have also been used.

### 4.3. Microorganisms and Culture

*E. coli* ATCC 25922, K12 (MG1655 or K12 5K), D21, and D21f2 were stored in Müller Hinton Broth (MHB, Carl Roth, Karlsruhe, Germany) supplemented with 20% (*v*/*v*) glycerol at −80 °C. Overnight cultures have been made from single colonies by incubating the cells at 37 °C and 200 rpm in MHB. The main culture was made with an inoculum of 0.05 or 0.01 OD_600_. The cultures were grown until the mid-logarithmic phase which was reached after 3.5–4 h.

### 4.4. Antimicrobial Activity

Antimicrobial activity was assessed as previously described [11]. Briefly, *E. coli* strains were cultured to mid-logarithmic phase in MHB at 37 °C under shaking conditions (200 rpm) and washed once with sodium phosphate buffer (PBS, 20 mM NaH_2_PO_4_/Na_2_HPO_4_, 130 mM NaCl, pH 7.4). Approximately 1 × 10^6^ CFU/mL in PBS (calculated from the absorbance of the suspension at 600 nm) were incubated with OP-145 (final concentrations: 0–6.4 μM) and SAAP-148 (final concentrations: 0–1.6 μM) for 2 h at 37 °C under shaking conditions (300rpm). Thereafter, the number of viable bacteria was determined by plating 10-fold serial dilutions of 100 µL sample on diagnostic sensitivity agar, Müller Hinton Agar (MHB+2 w% Agar). The same experiment was also performed in a time-dependent way, where the antimicrobial activity was determined after 5, 10, and 20 min for OP-145 and 1, 2, 3, 4, and 5 min for SAAP-148. Antimicrobial activity is expressed as the 99.9% lethal concentration (LC_99.9_), i.e., the lowest peptide concentration that killed ≥99.9% of bacteria within 2 h [14,19,32]. Alternatively, bacteria were incubated with peptides for 5 min in pure PBS before MHB was added and then, bacterial growth was observed in the Bioscreen for 24 h at 420–560 nm. The concentration of the peptide that completely diminished growth of bacteria was used as the minimal inhibitory concentration (MIC). The results are average results of at least three or more experiments.

### 4.5. Zeta Potential Measurements

For the zeta potential measurements, mid-logarithmic growing cells were diluted to 1 × 10^7^ CFU/mL in Hepes buffer (10 mM Hepes, 140 mM NaCl, pH 7.4) and treated with indicated peptide concentration. Upon incubation time of 5 min at room temperature, suspensions were injected to the zeta cuvettes and measured on a Zetasizer (Zetasizer NANO, Malvern Instruments, Herrenberg, Germany) according to methods published before [14,17,18,19]. Experiments were performed in presence or absence of 0.2 mM EDTA, but this did not affect the measurements.

### 4.6. Lipid Staining of Bacteria by Fluorescence Microscopy

The lipid staining of bacteria was performed similar to previous protocols [17,19,26]. A mid-logarithmic growth-phase culture was diluted in PBS to 2.5 × 10^8^ CFU/mL. 200 µL of these cell-suspension were incubated with peptide concentrations 2–3 times lower than their LC_99.9%_ for 20 min at 37 °C and 300 rpm, before being stained with 10 µg/mL Nile Red (Sigma Aldrich, Steinheim, Germany), a fluorescent membrane dye. Nile Red stock solution of 1 mg/mL was prepared in methanol (Carl Roth, Karlsruhe, Germany). After centrifugation, 1 µL of the cell pellet was mounted onto a microscope slide with agar (2 w%) and covered with a coverslip of 0.17 µm thickness (Menzel, Inc., Spartanburg, SC, USA). 

Microscopy was performed using a Leica SP5 confocal microscope (Leica Microsystems, Inc., Singapore) with spectral detection and a Leica HCX PL APO CS 63x NA 1.4 oil immersion objective. Nile Red was excited at 561 nm and fluorescence emission was detected between 570–750 nm. A 488 nm laser line was additionally activated for simultaneous acquisition of fluorescence and transmission data. Images were recorded using 47 × 47 nm sampling (x/y).

### 4.7. Membrane Depolarization Assay and Measurement by Fluorescence Spectroscopy

The assay was performed similarly to our previous protocol [19]. Cells in mid-logarithmic phase were collected to a concentration of 1 × 10^7^ CFU/mL and stained with 1 µM DiSC3(5) for 30 min in Hepes buffer (10 mM Hepes, 140 mM NaCl, pH 7.4) containing 100 mM KCl to allow self-assembly of the dye and equilibration of K^+^ ions. Peptides were added at 0.4 µM and the DiSC3(5) fluorescence increase followed at 622/685 nm in a VARIAN Cary Eclipse Fluorescence Spectrophotometer. Notably, higher peptide concentrations did not result in higher fluorescence.

### 4.8. Membrane Permeabilization Assay Using Flow Cytometry 

Fluorescence-activated cell sorting (FACS) experiments were performed in analogy to previously published protocols ([18,19]. Bacteria were cultured to mid-logarithmic phase at 37 °C under vigorous shaking and then washed once with NaPi-buffer (5 min, 5000 g). This bacterial suspension was diluted in PBS to 1 × 10^6^ CFU/mL and stored on ice. A total of 600–1200 µL of the cell suspensions were incubated for five minutes at room temperature with propidium iodide (PI, 1 µg/mL final concentration in sample) in the dark. Afterwards, the sample was transferred in polystyrene-tubes and PI fluorescence was measured with BD LSR Fortessa^TM^ in real-time using the BD FACSDiva Software Version 8.0.1. Selective gating was implemented to distinguish between PI-positive cells (with permeabilized membranes) and PI-negative cells (with intact membranes) within the cell populations. The scan rate involved utilizing an average of 300–400 events per second. The gating strategy utilized in this study is illustrated in a previously published manuscript by Malanovic et al. [17]. After approximately 30 s, defined peptide solutions (with final concentrations of 1.6–12.8 µM OP-145 and 0.4–1.6 µM SAAP-148) were added to the labeled bacterial cells, mixed and fluorescence of PI was followed for 20 min for OP-145 and 5 min for SAAP-148. As a control, the PI fluorescence of bacteria without peptide was measured.

### 4.9. Preparation of Liposomes

For DSC analysis, lipid films of 1 mg were prepared from 10 mg/mL stock solutions of single lipids. Lipid mixtures, e.g., DPPG/DPPE (25/75), DPPG/DPPE/TMCL (20/60/10), POPG/POPE (25/75), POPG/POPE/TMCL (20/60/10) were evaporated under a stream of nitrogen and dried in vacuum overnight. The films were stored at 4 °C until use. 

Lipid vesicles were formed upon addition of PBS to the lipid films. In the case of peptide-containing vesicles, peptides had been dissolved in PBS to a final volume of 1 mL prior to adding to the lipid films (total concentration of 1 mg/mL). Formation of lipid vesicles was achieved by intermittent vigorous vortexing of the probes, which were incubated at a temperature above the phase transition of the respective phospholipids, in our case in a sand bath at 65 °C for one hour. 

For leakage measurements, liposomes with concentration of 20 mg/mL in fluorophore-buffer (10 mM Hepes, 68 mM NaCl, 12.5 mM ANTS, 45 mM DPX, pH 7.4) were used. LUVs of ca. 100 nm were obtained by extrusion of the hydrated liposomes by applying 20 cycles through a polycarbonate filter (Millipore-Isopore^TM^) of 0.1 µm pore size [32]. ANTS (8-aminonaphthalene-1,3,6-trisulfonic acid, disodium salt) and DPX (p-xylene-bis-pyridinium bromide) were purchased from Molecular Probes (Eugene, OR). The size of vesicles was measured using a Zetasizer (Zetasizer NANO, Malvern Instruments, Herrenberg, Germany).

### 4.10. DSC

DSC measurements were performed using a Microcal VP-DSC high-sensitivity differential scanning calorimeter (Microcal, Northampton, MA, USA). Scans of 1 mg/mL lipid were recorded at a constant rate of 30 °C/h and data were analyzed using Microcal’s Origin software [10]. Calorimetric enthalpies were calculated by integration of the peak areas after baseline correction and normalization to the mass of phospholipid. The phase transition temperature was defined as the temperature at the peak maximum.

### 4.11. Vesicle Leakage Assay

Leakage of the aqueous content of the 8-aminonaphthalene-1,3,6-trisulfonic acid/p-xylene-bis-pyridinium bromide (ANTS/DPX)-loaded liposomes composed of POPG/POPE, POPG/POPE/TMCL and *E. coli* lipid extracts (polar/total) upon incubation with the peptides OP-145 and SAAP-148 was determined as described previously [16]. Briefly, 20 mg/mL ANTS/DPX-loaded lipid vesicles of defined size were separated from free fluorescent dye by exclusion chromatography using a column filled with Sephadex^TM^ G-75 (Amersham Biosciences, Buckinghamshire, UK) fine gel swollen in an iso-osmotic buffer (10 mM Hepes, 140 mM NaCl, pH 7.4). The phospholipid concentration was determined by phosphate analysis as described above. Fluorescence emission from the ANTS/DPX loaded lipid vesicles were obtained at 37 °C at an excitation wavelength of 360 nm and an emission wavelength of 530 nm. The slit width of the excitation and emission monochromators was 10 nm. For the measurements, lipids with a final concentration of 50 µM were used. Fluorescence emission was recorded as a function of time before and after the addition of incremental amounts of peptide ranging from 0.125 up to 16 μM, corresponding to peptide-to-lipid molar ratios from 1:400 to 1:3. The measurements were performed on a VARIAN Cary Eclipse fluorescence spectrophotometer combined with Cary Eclipse Software (Scan). Percentage of leakage was calculated from the fraction of the leakage (*I_F_*) according to the following formula:

$$I_{F}=\frac{\left(F-F_{0}\right)}{\left(F_{max}-F_{0}\right)}\;$$

where *F* is the measured fluorescence, *F*_0_ is the initial fluorescence without peptide, and *F_max_* is the fluorescence corresponding to 100% leakage gained by addition of 20 µL 10% Triton X-100 [10,16].

## 5. Conclusions

To summarize the current study, the comparison of OP-145 and SAAP-148 revealed differences in their physicochemical properties and their effect on bacterial membranes. Despite having similar lengths, hydrophobicity, and amphiphathicity, SAAP-148 exhibited superior membrane permeability compared to OP-145. The larger hydrophobic region and presence of specific amino acid residues in SAAP-148 contributed to its enhanced membrane interaction. Additionally, the interaction with phospholipids such as CL and PE further enhanced the effectiveness of SAAP-148 in disrupting membranes. OP-145, on the other hand, demonstrated membrane irregularities and membrane damage without significant permeabilization. These irregularities progressively compromised membrane integrity, leading to cell death. The presence or absence of specific components of LPS did not significantly affect the antimicrobial activity of both peptides, suggesting that their primary killing mechanism is not importantly dependent on LPS composition. The binding interactions between the peptides and LPS, particularly the O-antigen, may contribute to bacterial surface neutralization and the peptides’ ability to modulate an immune response. Further studies are needed to fully understand the mechanism and contributions of these interactions, and to determine whether membrane depolarization and/or membrane permeabilization are general phenomena in peptide-induced bacterial killing.

## Figures and Tables

**Table 1:** Strains used in this study.

*E. coli* Strain	Relevant Genotype	Source/Reference
**Full length LPS producing strain**		
ATCC 25992	*wildtype*	LGC Standards GmbH, Germany
**K-12 derived strains lacking O-antigen**		
MG1655	*F-*, *λ-*, *ilvG*, *rfb-50*, *rph-1*	[36]
K-12 5K	*tre*, *thi*, *rpsl+*, *kdsR*, *kdsM+*, *lac*	[37]
**K12-derived strain with modifications in the outer core**		
D12	*F-*, *proA23*, *lac-28*, *tsx-81*, *trp-30*, *his-51*, *rpsL173(strR)*, *ampCp-1*	[38,39,40]
**D12-derived strain lacking O-antigen, outer and the inner core of LPS**		
D12f2	*F-*, *proA23*, *lac-28*, *tsx-81*, *trp-30*, *his-51*, *rpsL173(strR)*, *rfa-31*, *rfa-1*, *ampCp-1*	[38,39,40,41]

**Table 2:** Antimicrobial activity of SAAP-148 and OP-145 against *E. coli* producing varying lengths of LPS. Growth of bacteria was monitored using optical density measurements and colony counting on agar plates in the presence peptide concentrations ranging from 0.2–12.8 µM. The minimal inhibitory concentration (MIC) and lethal concentration (LC) were determined to quantify the antimicrobial effectiveness of the peptides. based on their minimal inhibitory concentration (MIC) or lethal concentration (LC). The MIC represents the lowest concentration of the peptides that inhibits visible growth of the bacteria. On the other hand, the LC corresponds to the concentration at which the peptides are capable of killing 99.9% of bacterial cells. The results represent ranges of at least three or more independent experiments. Where no range is indicated, MIC and LC_99.9%_ were identical in all experiments.

		Antimicrobial Activity (µM)			
Peptides		ATCC25992	K12	D21	D21f2
SAAP-148	MIC	0.8	0.8	0.8	0.8
OP-145		3.2	1.6–3.2	1.6–3.2	1.6
SAAP-148	LC_99.9%_	0.4–1.6	0.2–0.4	0.2–0.4	0.4
OP-145		3.2–12.8	1.6–3.2	1.6–3.2	1.6–12.8

**Table 3:** Thermodynamic parameters (phase transition temperature (*T*_m_), half-width of transition (ΔT_1/2_) and corresponding enthalpies of POPG/POPE, POPG/POPE/TMCL, DPPG/DPPE, and DPPG/DPPE/TMCL in presence and absence of SAAP-148 and OP-145. Parameters are shown for heating and cooling scans recorded for 1 mg/mL membrane at lipid-to-peptide molar ratio of 25:1. Data are the representative results of three independent experiments with standard deviations less than 5%.

	Heating Scans			Cooling Scans		
	Enthalpy * [kcal/mol]	*T*_m_ [°C]	∆*T*_1/2_ [°C]	Enthalpy * [kcal/mol]	*T*_m_ [°C]	∆*T*_1/2_ [°C]
**POPG/POPE**	5.8	21.1	2.4	5.3	18.7	1.4
**+OP-145**	6.4	18.9	3.3	0.6 3.5 2.0	9.5 16.5 19.2	3.9 0.9 0.6
**+SAAP-148**	1.5 4.5	15.1 20.9	4.0 3.4	0.9 1.8 2.9	12.0 17.0 19.0	3.1 0.6 1.2
**DPPG/DPPE**	9.2	60.4	1.6	7.9	59.3	0.4
**+OP-145**	7.5	60.6	1.9	1.0 5.9	52.4 59.8	2.9 0.2
**+SAAP-148**	0.9 3.0	55.1 60.3	1.9 1.7	0.6 2.9	52.4 59.7	4.6 0.3
**POPG/POPE/TMCL**	7.6	26	6.2	5.7	25.4	2.6
**+OP-145**	7.2	26.4	6.2	7.3	25.1	5.6
**+SAAP-148**	1.4	24.4	5	1.6	24.6	4.1
**DPPG/DPPE/TMCL**	11.1	56.5	3.4	10.5	55.9	1.1
**+OP-145**	9.5	59.6	2.2	0.7 8.8	39.0 58.9	8.5 1.0
**+SAAP-148**	1.2 4.6	49.5 55.9	6.1 5.5	5.0	56.7	5.0

* In cases where peaks overlapped, total enthalpy was reported in the table.

## Data Availability

Data are available upon request by corresponding author.
